# Brucella Seropositivity at a Tertiary Care Hospital in Chandigarh

**DOI:** 10.7759/cureus.58711

**Published:** 2024-04-22

**Authors:** Uneza Husain, Sunil Sethi, Rakesh Yadav

**Affiliations:** 1 Department of Medical Microbiology, Postgraduate Institute of Medical Education and Research, Chandigarh, IND; 2 Department of Microbiology, Government Doon Medical College, Dehradun, IND

**Keywords:** serology, brucella, sat, neglected tropical disease, brucellosis

## Abstract

There are reports that the neglected zoonotic tropical disease brucellosis is reemerging today. Serological tests are being widely used in the diagnosis of brucellosis. In the present study, we performed a standard agglutination test (SAT) on 1348 suspected cases of brucellosis during the period of four years from April 2018 to March 2022. We noticed an increase in seropositivity from 2.6% in the year 2018-19 to 7.4% in the year 2021-22. We also noticed a spike in seropositivity in the years 2019-20 (12.5%). Our study shows the recent trend in seropositivity of the disease in Chandigarh and, hence, can be a meaningful addition to the existing serological diagnostic data related to brucellosis.

## Introduction

Named after David Bruce, *Brucellae* are fastidious, gram-negative coccobacilli responsible for causing a neglected zoonotic tropical disease called brucellosis. Although the various species of the genus *Brucella include B. abortus, B. suis, B. ovis, B. melitensis, B. canis, B. neotomae, B. pinnipedialis, B. ceti, B. microti*, and *B. inopinata*, the disease is mainly caused by *Brucella melitensis, B. suis, B. abortus*, and rarely by *B. canis* in human beings [1]. The various modes of transmission of *Brucella* are consuming unpasteurized milk or other raw dairy products, inhalation, or a breach in the skin or mucous membrane after contact with infected animals [2]. Hence, hunters and those who work at the slaughterhouse are more at risk. This information should be utilized to educate the masses, as a considerable knowledge gap still exists regarding the risk factors for the disease. The presenting features of human brucellosis include fever (remittent), sweating, headache, anorexia, hepatosplenomegaly, and/or musculoskeletal symptoms [2]. Complications such as pneumonia, endocarditis, meningoencephalitis, and pyelonephritis can also occur [1,2]. *Brucellae* show tropism for the reticuloendothelial system and have a special predilection for the placenta, as erythritol present in the placenta enhances their growth. The various diagnostic options available are blood culture, Rose Bengal test, standard agglutination test (SAT), 2-mercaptoethanol (2-ME) test, enzyme immunoassay (EIA), latex agglutination test, lateral flow immunochromatography (for IgM and IgG detection), and polymerase chain reaction (PCR) [3-5]. Although widely used in diagnosing brucellosis, all the serologic tests have to be interpreted with caution in light of clinical data and the context of the local prevalence of brucellosis [1]. Our study provides insight into the recent trend of brucellosis seropositivity in Chandigarh, offering a valuable contribution to the existing serological diagnostic data on this disease.

## Materials and methods

Study design

In the present study, we performed retrospective data analysis related to the seropositivity of 1348 suspected patients of brucellosis presenting to our hospital based on SAT during the period of four years from April 2018 to March 2022.

Study setting

The study was conducted at the Postgraduate Institute of Medical Education and Research (PGIMER), a tertiary care center located in Chandigarh, India. The medical records of patients who visited the hospital during the study period were retrospectively reviewed.

Data collection

Electronic medical records (EMRs) were accessed to identify patients who underwent serological testing for *Brucella* antibodies during their hospital visit. The data extracted included demographic information (age, gender) and laboratory results.

Inclusion criteria

Patients of all ages who underwent serological testing for *Brucella* antibodies during the study period.

Patients with available medical records containing relevant demographic and laboratory testing information.

Exclusion criteria

Patients with incomplete medical records or missing key information.

Procedure

Serum specimens collected in a BD vacutainer plastic tube were used to perform SAT. Sera were tested for the highest dilution at which 50% agglutination was observed using an antigen (*Brucella abortus* S99) obtained from the Indian Veterinary Research Institute, Izatnagar, Uttar Pradesh. The degree of agglutination was judged by the opacity of the supernatant fluid in the test tubes as compared to the antigen control tubes (as per the antigen manufacturer’s instructions). The endpoint titer of 80 IU/mL or more was considered significant. To differentiate between IgM and IgG antibodies, we also performed the 2-ME test on all seropositive sera. Age and sex distribution related to seropositivity were also assessed.

Data analysis

For statistical analysis, demographic and corresponding laboratory investigation data were entered in a Microsoft Excel (Microsoft Corporation, Redmond, Washington, United States) spreadsheet. The data were analyzed using the Chi-square test with the help of IBM SPSS Statistics for Windows, Version 23 (Released 2015; IBM Corp., Armonk, New York, United States) The statistical significance threshold was set at .05 p-value.

## Results

**Figure 1:**
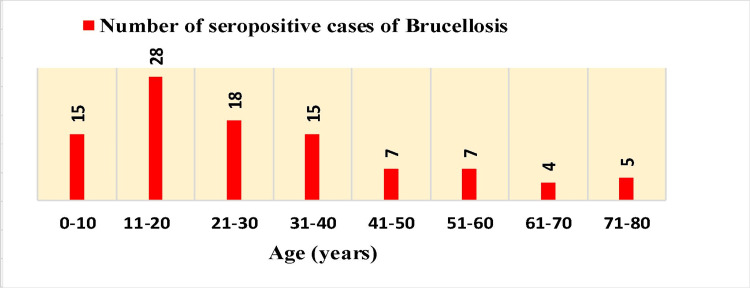
Age-wise distribution of seropositive cases of brucellosis

Out of 1348 patients, 99 (7.3%) were found to be seropositive for brucellosis, with ages ranging from four to 78 years. The year-wise distribution of data related to the disease from April 2018 to March 2022 (four years) has been summarized in Table 1. It can be derived from the table that there was a sharp increase in the number of seropositive cases during the period of April 2019-March 2020 in comparison to April 2018-March 2019, followed by a fall in the seropositivity rate during April 2020-March 2022, but the figures were still higher as compared to April 2018-March 2019 data. Overall, we noticed a statistically significant increase in *Brucella* seropositivity from 12 (2.6%) in the year 2018-19 to 22 (7.4%) in the year 2021-22 (p-value = 0.00179). We found a slightly greater number of males 57 (57.6%) to be seropositive for brucellosis as compared to females 42 (42.4%), with the male-to-female ratio being 1.36:1. The age-wise distribution of data from April 2018 to March 2022 showed maximum *Brucella* seropositivity in the age group of 11-20 years, followed by 21-30 years, as shown in Figure 1. Based on the 2-ME test, significant titers of IgG antibodies were detected in 41 seropositive cases of brucellosis. 

**Table 1:** Year-wise distribution of data related to brucellosis from April 2018-March 2022 (four years) based on SAT N: number; SAT: standard agglutination test

Duration (years)	Total tested (N)	Total seropositive (N(Percentage))
April 2018-March 2019	463	12 (2.6%)
April 2019-March 2020	417	52 (12.5%)
April 2020- March 2021	170	13 (7.6%)
April 2021-March 2022	298	22 (7.4%)
Total	1348	99 (7.3%)

## Discussion

Brucellosis is neglected in many countries around the world, causing substantial costs in endemic places. Brucellosis is estimated to be responsible for 500,000 cases in humans every year globally [6]. As the disease is underreported, the exact incidence is difficult to predict. The disease is endemic in South and Central America, Mexico, Africa, the Indian subcontinent, Asia, the Mediterranean Basin, and the Middle East [7]. Alam et al. documented 7% (28/400) positivity for brucellosis by *Brucella*-specific latex agglutination test and found 22 (5.5%) out of 400 samples to be *Brucella* genus-specific real-time PCR positive [3]. Pathak et al. tested serum samples from pyrexia of unknown origin (PUO) cases and occupationally exposed individuals for the detection of brucellosis. They found 4.25%, 3.54%, 6.02%, and 4.96% of samples as positive by the Rose Bengal plate test (RBPT), serum agglutination test, indirect enzyme-linked immunoassay (ELISA), and IgG ELISA, respectively [8]. In our study, we found similar results with overall seropositivity during 2018-2022 at 7.3% (99/1348) by SAT. We also detected a higher number of males with *Brucella* seropositivity (57.6%) as compared to females (42.4%) in our study. The possible reason is that males were more exposed to constant close contact with livestock during outdoor agricultural activities than the women in our study, similar to the study done by Yuan et al. [9]. We detected maximum *Brucella* seropositivity in the age group of 11-20 years, followed by 21-30 years. The various factors attributed to this finding in teenagers and young adults are engagement in high-risk occupations (e.g., agriculture), outdoor activities (e.g., camping, hiking, and hunting) increasing exposure to infected animals, and social behaviors involving consumption of unpasteurized dairy products or undercooked meat. These factors collectively heighten susceptibility to the disease in this age demographic.

Buchanan and Faber reported that a negative 2-ME test is ''strong evidence'' against chronic brucellosis [10]. Our study detected 58.6% (58/99) seropositive sera on SAT with a negative 2-ME test. The SAT is considered one of the most economical and, hence, widely used tests. The primary limitation of serological detection of brucellosis is the lack of discriminatory power to differentiate between *Brucella* species and biovars, as well as cross-reactivity with other gram-negative bacteria [11]. The added advantage of ELISA over SAT is that it can be used to test a larger number of samples, and the turnaround time is also less. In a comparative study of *Brucella* SAT and ELISA done by Memish et al. in patients with *Brucella* bacteremia, the sensitivity and specificity of the SAT were 95.6% and 100.0%, respectively, while those of the ELISA IgG were 45.6% and 97.1%, and those of the ELISA IgM were 79.1% and 100.0%, respectively [5]. The combination of doxycycline with rifampin or an aminoglycoside is effective in the treatment of brucellosis in humans. A retrospective study, such as this one, provides useful information regarding the trend of disease that can help formulate policies and strategies for the control and eradication of this occupationally hazardous disease, which is otherwise neglected [12,13]. 

Brucellosis control necessitates a One Health approach, recognizing the interconnection between human, animal, and environmental health [14]. Efforts to control the disease should involve collaboration between veterinary and medical professionals, as well as environmental and wildlife experts. This integrated approach is essential for comprehensive surveillance, prevention, and control measures. Improving the diagnostic capacity for brucellosis in both humans and animals is essential for early detection and timely intervention. Access to accurate and reliable diagnostic tests, including serological assays, bacterial culture, and molecular techniques, is critical for surveillance and control efforts. Investments in laboratory infrastructure and the training of laboratory personnel are needed to enhance diagnostic capabilities, particularly in resource-limited settings.

Limitations of the study

The limitation of the present study is its retrospective nature; thus, clinical correlation could not be established. The present study relied on data collected from medical records, which may not capture all cases of brucellosis within our study population. Moreover, patients with mild or asymptomatic infections are less likely to visit hospitals or be diagnosed, leading to the underrepresentation of these cases in the study population. This selection bias can skew the results and compromise the generalizability of the findings. Nonetheless, future researchers can use the results of the present study as a baseline for further investigation in this regard. 

## Conclusions

The disease brucellosis is said to be re-emerging nowadays, and hence its diagnostic evaluation plays an important role in patient management. Hence, looking at the recent trend with an increase in seropositivity, clinicians should keep it as one of the differentials in the diagnosis of patients with pyrexia of unknown origin. In conclusion, the present study emphasizes the need for increased awareness regarding timely diagnosis, enhanced laboratory detection, and improved brucellosis control measures such as vaccination of animals, safe handling of animals or their tissues, and accelerated surveillance of the disease.
